# Supplementary material for: Characterization of a pluripotent stem cell-derived matrix with powerful osteoregenerative capabilities
Source: Nat Commun. 2020 Jun 15;11:3025. doi: 10.1038/s41467-020-16646-2 (PMC7295745; doi:10.1038/s41467-020-16646-2)
Supplement: Supplementary file 2 — Supplementary Information [file 41467_2020_16646_MOESM2_ESM.pdf]

## Supplementary Figure 1

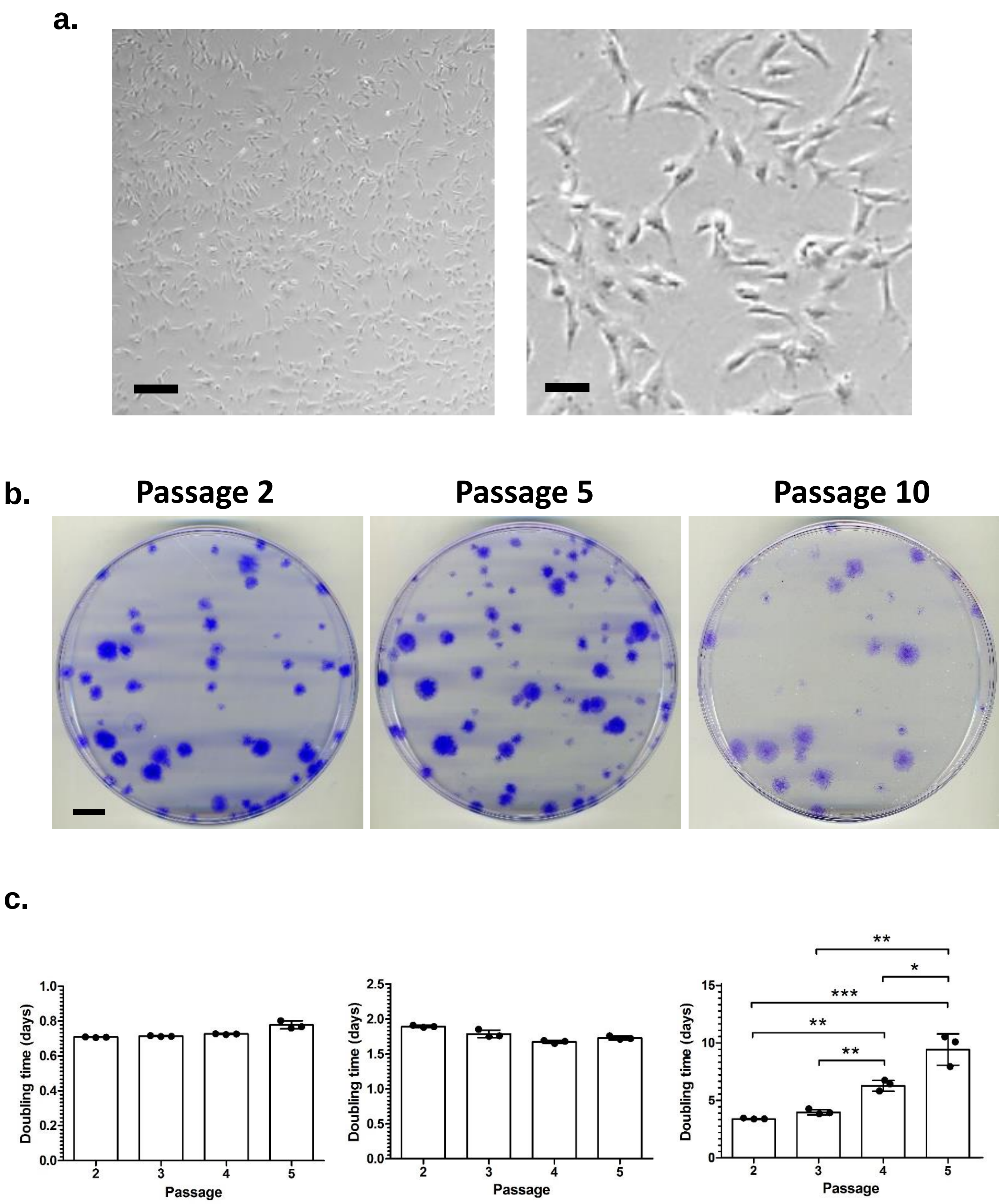


**Supplementary Figure 1: Morphology and proliferative capacity of ihMSCs:** Panel a: phase-contrast micrographs of ihMSCs on monolayer (bar, left = 250 microns, bar, right = 25 microns). Panel b: images of colonies generated from ihMSCs. Lighter crystal violet stain indicates reduced capacity to form dense monolayers at passage 10 (bar = 10 mm). Panel c: average doubling time in a single culture seeded at 500 cells per cm2 and allowed to proceed for 10 days. Results are presented for the early log phase (day 1-3, left), mid log phase (day 4-6, center) and late log to stationary phase (day 8-10, right). Statistics: data are presented as means with standard deviations. The data were compared using ANOVA with Tukey's post-test. * = $P<0.05$, ** = $P<0.01$, *** = $P<0.005$, n=4. Source data are provided as a Source Data file.

**Supplementary Figure 2**

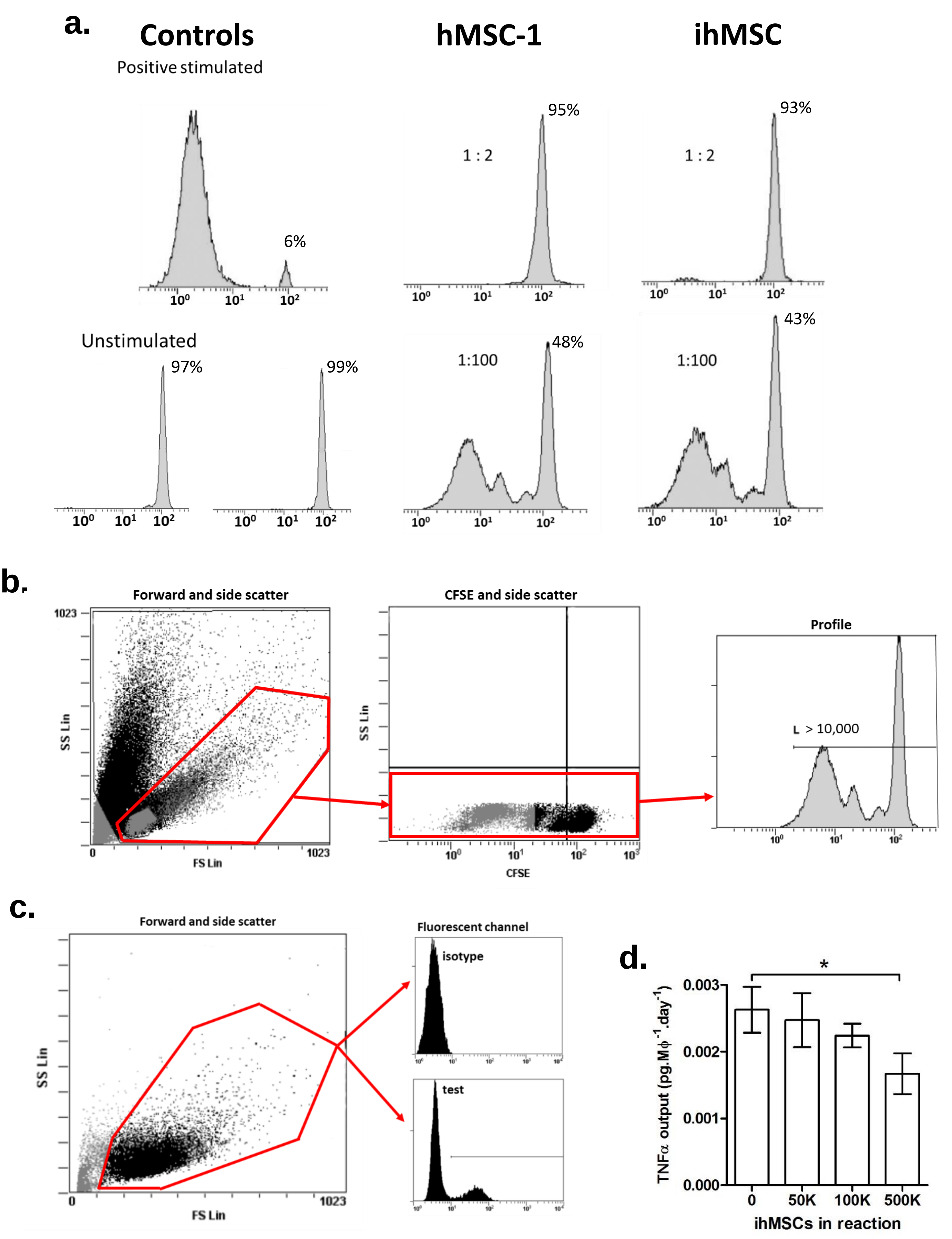


**Supplementary Figure 2: Immunomodulatory capacity of ihMSCs:** Panel a: mixed lymphocyte stimulation assayed after 5 days demonstrates that ihMSCs inhibit CFSE-labeled lymphocyte proliferation as measured by proliferative dilution of CFSE by flow cytometry. Controls on right are mixed PBLs stimulated in the absence of MSCs (above) and unstimulated PBL donors cultured alone (below). Assays performed in the presence of bone marrow derived hMSC donor-1 (center) and ihMSCs (right) are presented at a ratio of 1:2 (above) and 1:100 (below) MSC:mixed PBL. Panel b: gating strategy for CFSE-lymphocyte proliferation assays. Panel c: gating strategy for immunophenotyping of MSCs. Panel d: co-cultured ihMSCs inhibit secretion of TNFalpha by macrophages challenged with LPS. Source data are provided as a Source Data file.

## Supplementary Figure 3

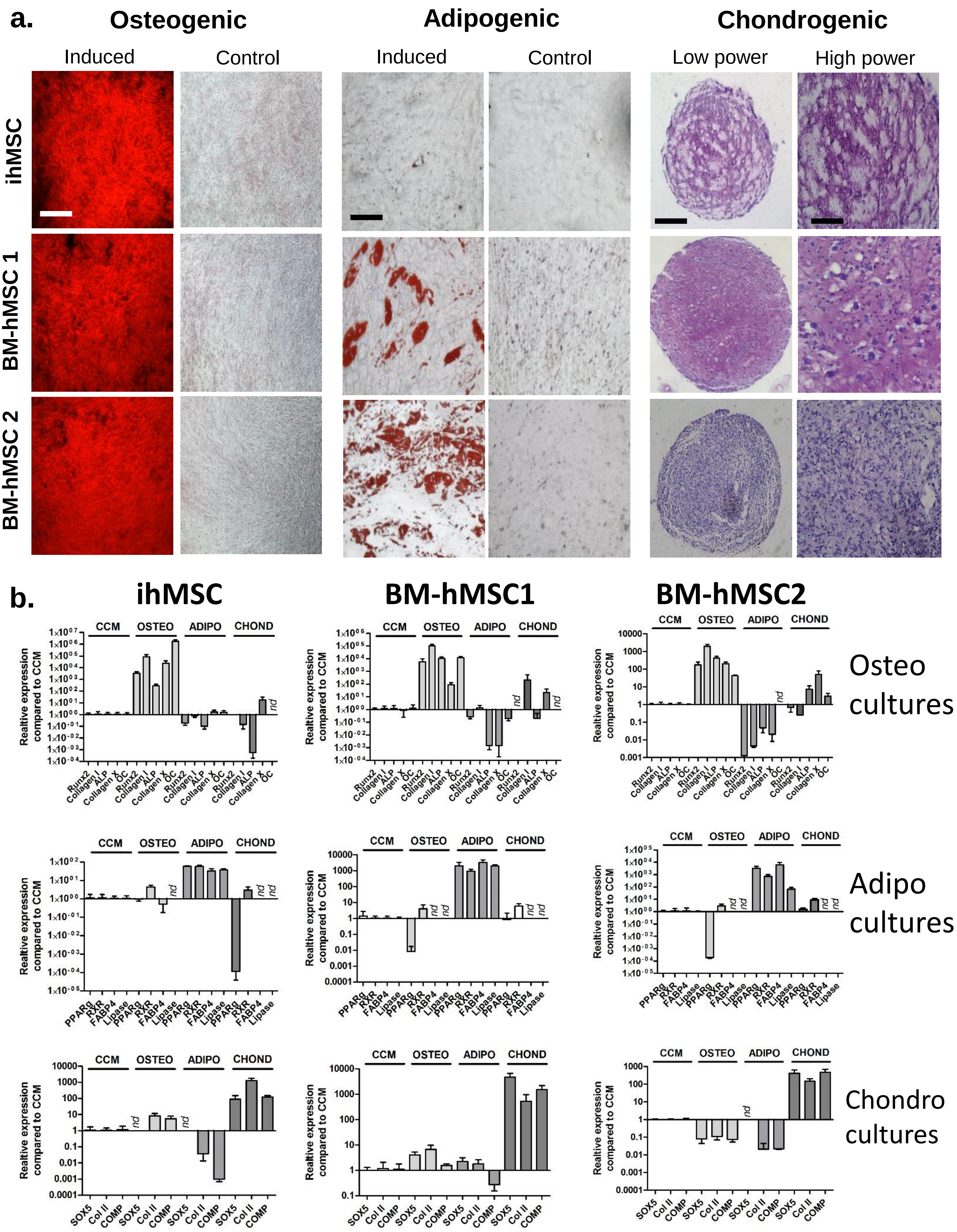


Legend on next page, Supplementary Figure 3C.

## Supplementary Figure 3 continued

**c.**

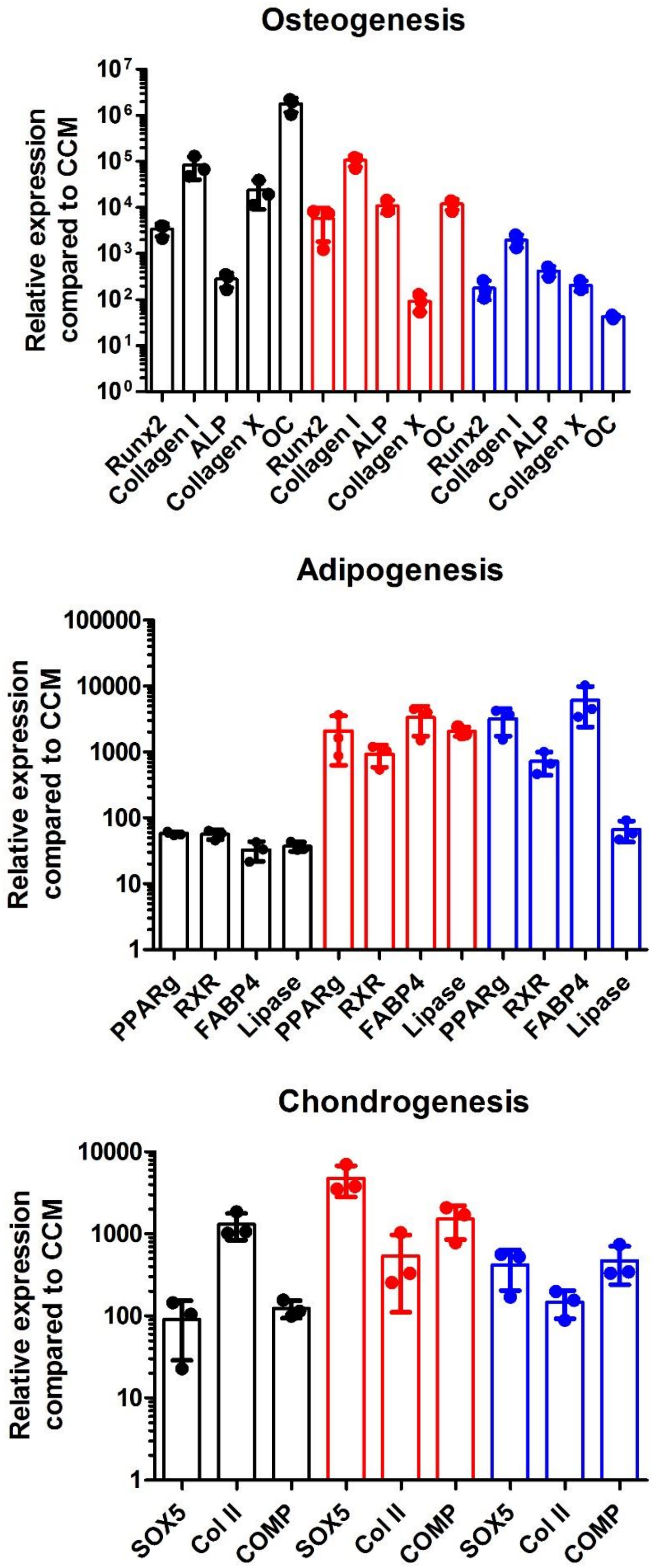


**Supplementary Figure 3: Differentiation capacity of ihMSCs compared to bone marrow derived hMSCs:** Panel a: standard osteogenic (left) adipogenic (center) and chondrogenic (right) assays on ihMSCs and two bone marrow derived hMSC preparations with high (BM-hMSC1) and low (BM-hMSC2) osteogenic capacity. For osteogenic and adipogenic assays, monolayers were incubated in differentiation media for 21 days followed by alizarin red S or oil red O staining respectively (bar = 200 microns). Controls lacked differentiation supplements. For chondrogenic assays, micromass pellets were incubated in chondrogenic media for 21 days followed by histological sectioning and toluidine blue staining. Low power (left, bar = 250 microns) and high power (right, bar = 50 microns) micrographs are presented. Sulphated proteoglycans indicative of cartilage stains purple. Panel b: quantitative PCR results used to generate heat maps in Fig1f. Cultures were exposed to standard differentiation assays and subjected to panels of biomarkers corresponding to osteogenesis, adipogenesis and chondrogenesis (x-axis). All fold changes are compared to complete culture media (CCM) containing no differentiation supplements. Assays generating signal below detectable limits are annotated with nd. Panel c: data from panel b plotted to facilitate comparison between MSC preparations. For panel b and c, data are presented as means (n=3) with SD (error bars). Source data are provided as a Source Data file.

## Supplementary Figure 4

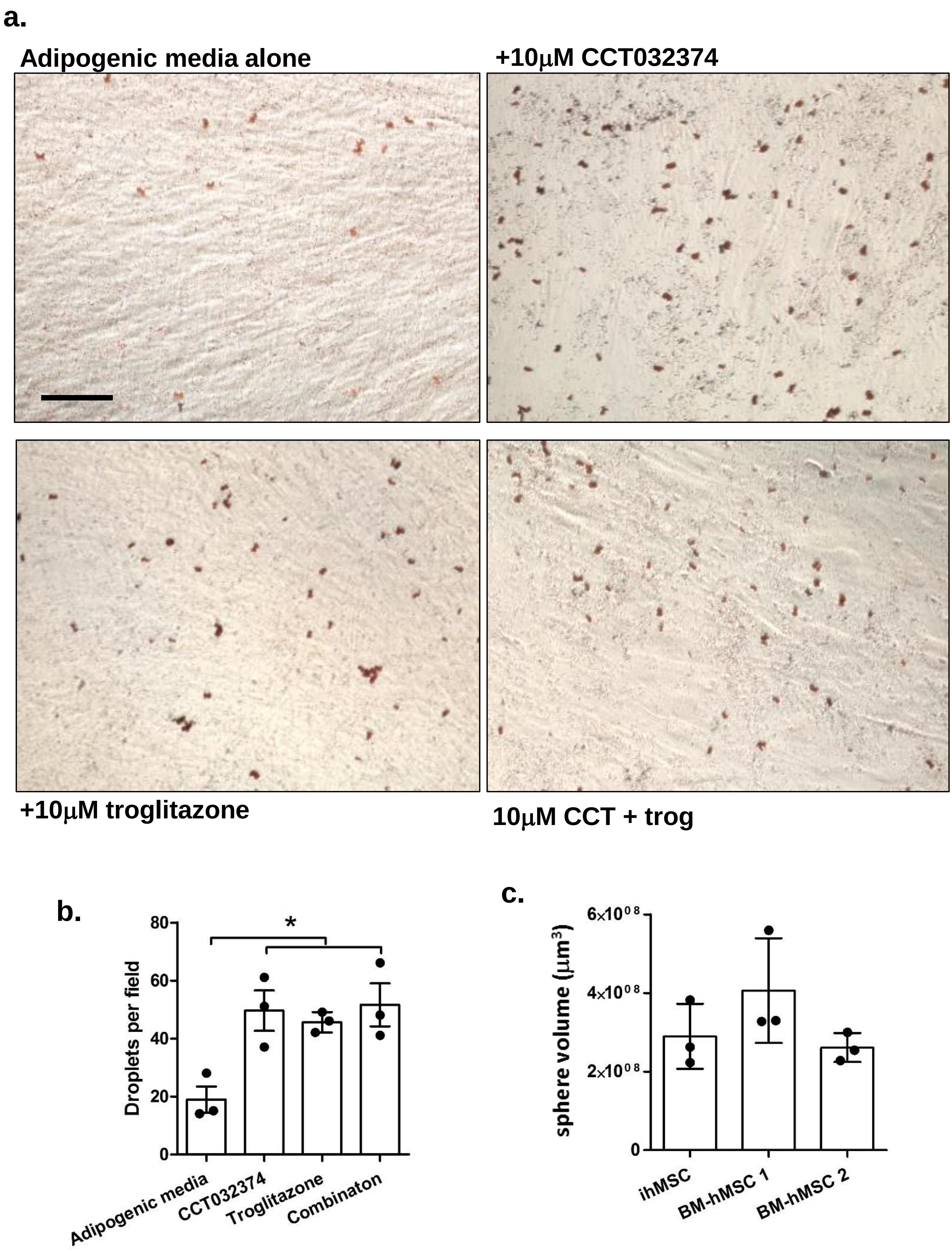


**Supplementary Figure 4: Differentiation capacity of ihMSCs:** Panel a: monolayers of ihMSCs were incubated under adipogenic conditions with the beta-catenin inhibitor (CCT032374), PPARgamma agonist troglitazone (trog), both, or vehicle (DMSO). Fat droplet clusters were stained with oil red O (bar = 200 microns). Panel b: counts of oil red O-stained lipid droplets per 10x field calculated as the mean of 5 random fields in 3 independent cultures per condition. Panel c: calculated volumes of chondrogenic micromasses generated from ihMSCs and BM-hMSC preparations. Statistics: For panel b and c, data are presented as means with SD (n=3) and were compared using one-way ANOVA with Tukey's post-test ($P<0.05$ = *). Source data are provided as a Source Data file.

**Supplementary Figure 5**

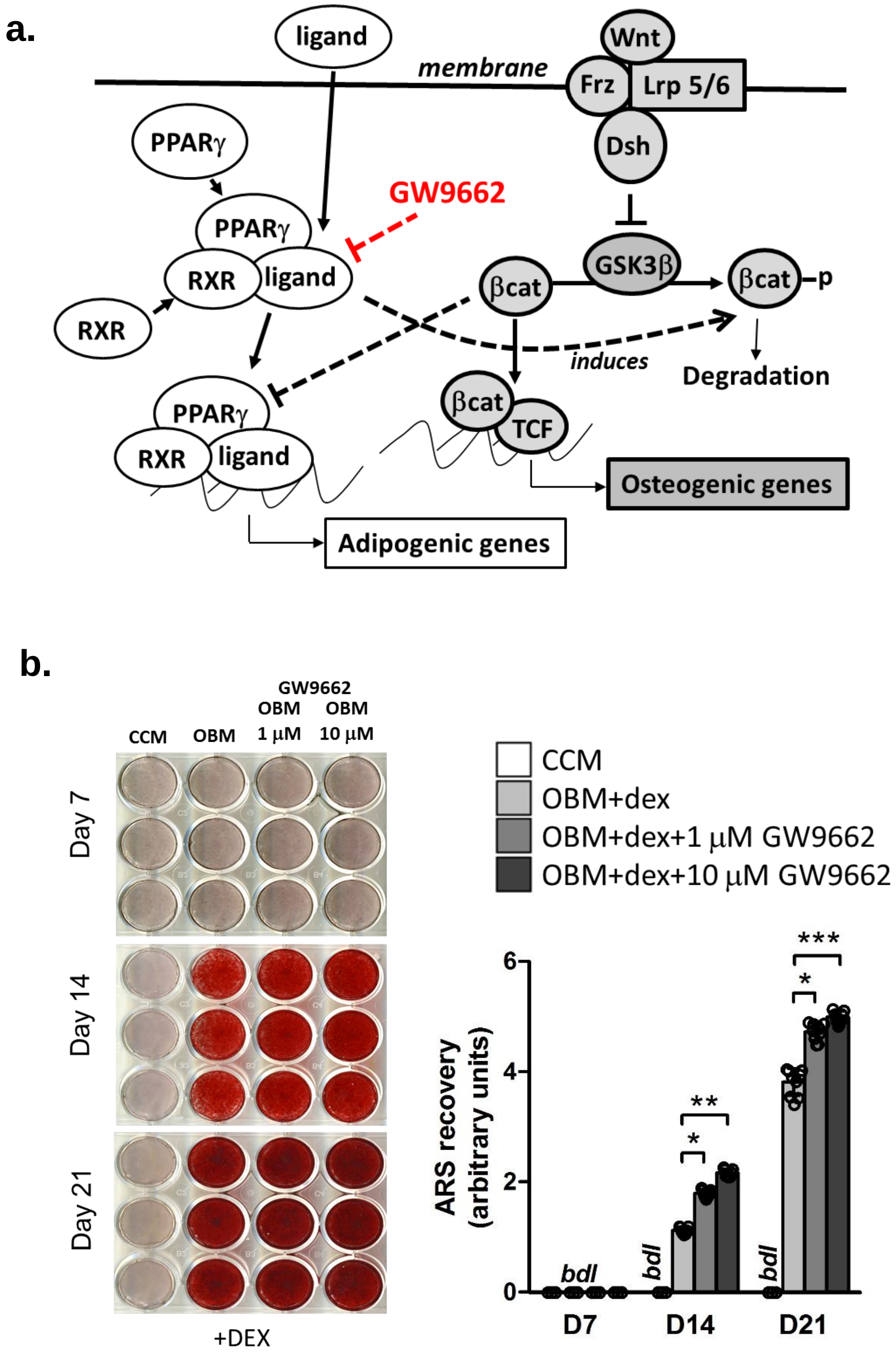


**Supplementary Figure 5: The effect of GW9662 on osteogegesis by OEihMSCs:** Panel a: diagrammatic representation of the co-inhibitory relationship between cWnt and PPARgamma axes. Panel b: mineralization of ihMSCs in the presence of OBM containing dexamethasone and doses of GW9662. Representative ARS-stained monolayers are presented (left) with quantification of staining (right). bdl: below detectable limits. Statistics: data are presented as means with standard deviations. The data were compared using one-way ANOVA with Tukey's post-test. * = P<0.05, ** = P<0.01, *** = P<0.005, n=6. Source data are provided as a Source Data file.

# Supplementary Figure 6

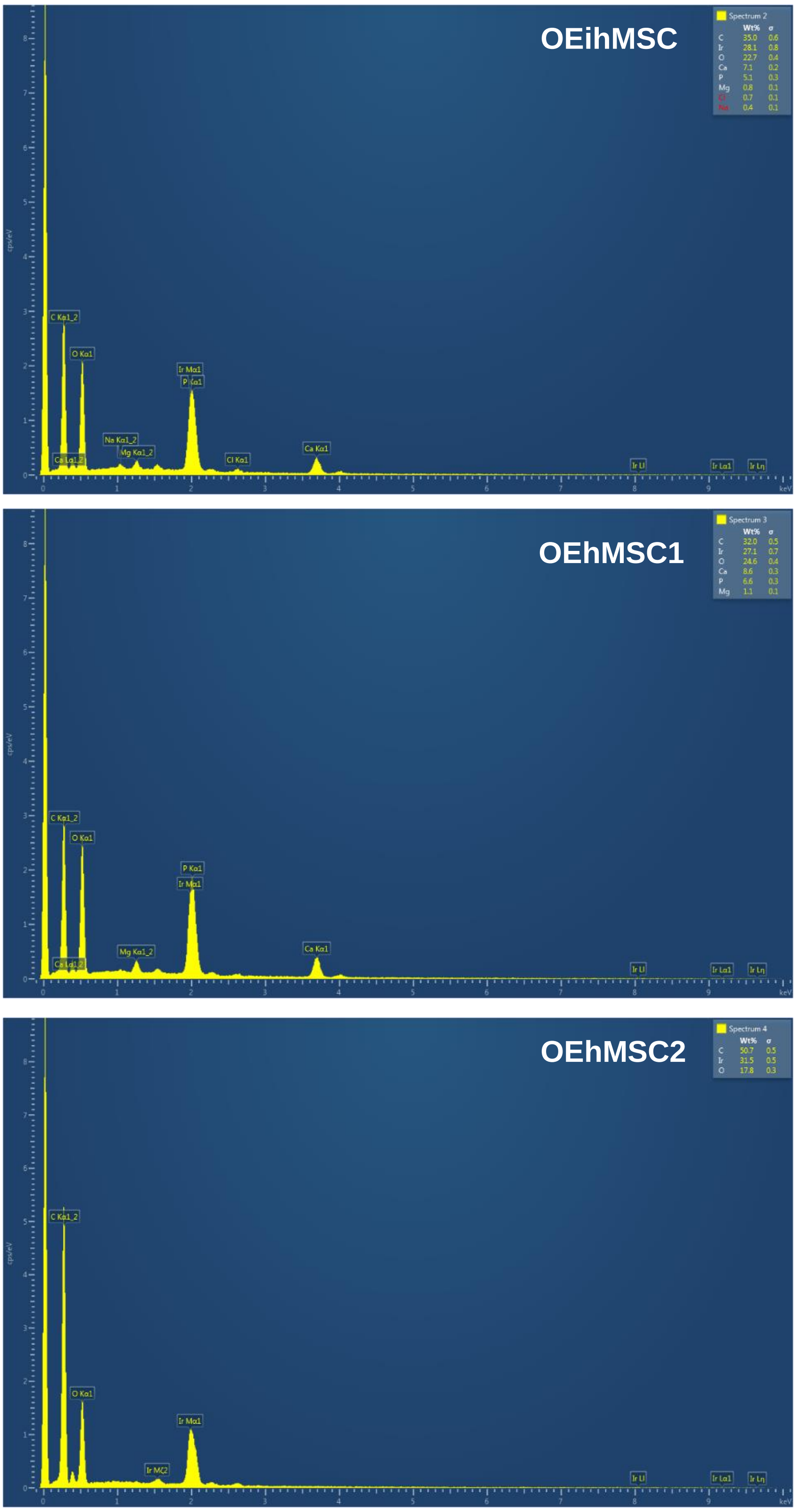


**Supplementary Figure 6: Raw EDM data from Fig3d.**

## Supplementary Figure 7

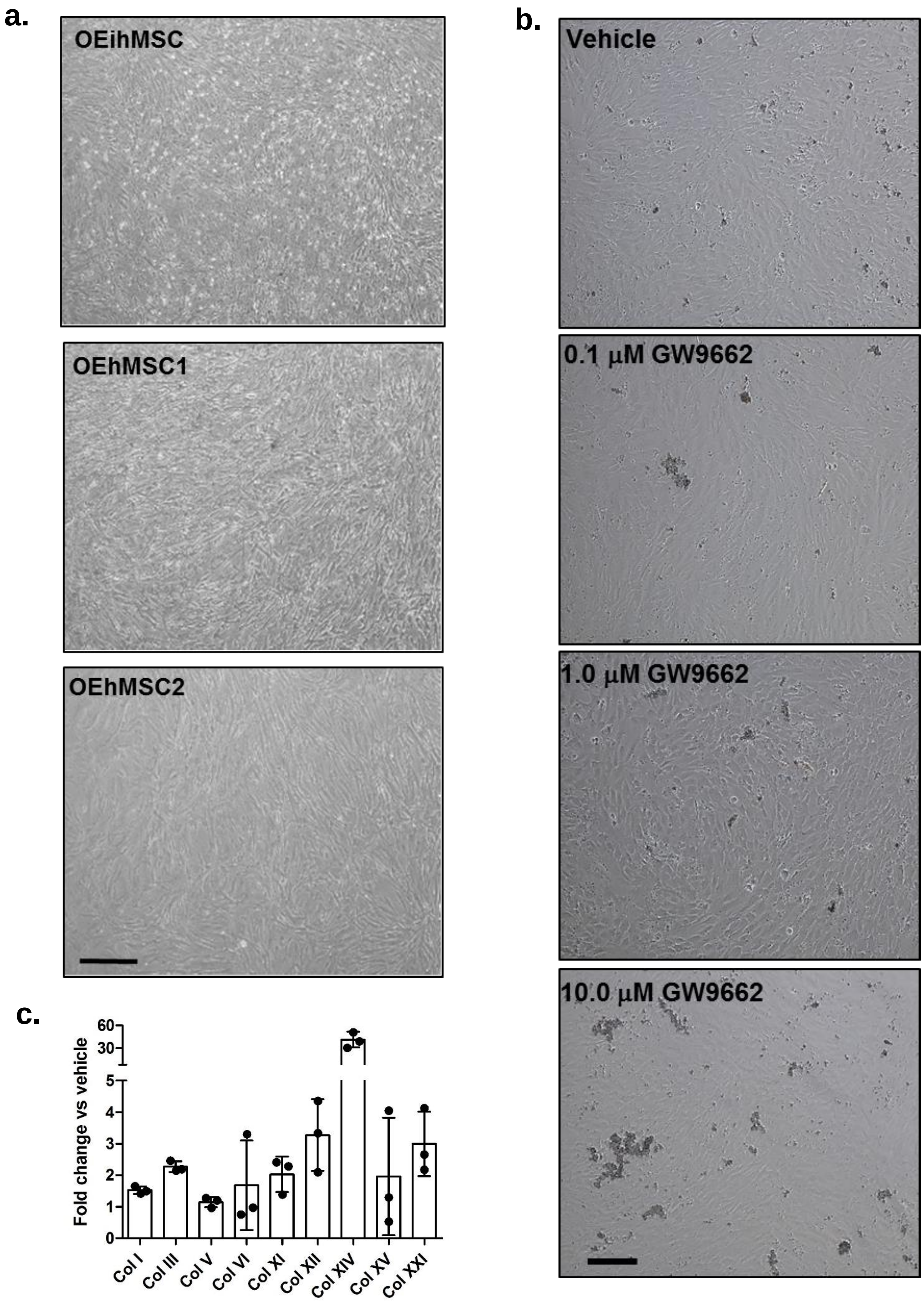


**Supplementary Figure 7: Deposition of ECM particles by osteogenic OEhMSCs and ihMSCs:** Panel a: monolayers of ihMSCs and BM-hMSCs were incubated in the presence of osteogenic base media with 10 micromolar GW9662. Refractile ECM particles are evident on osteogenic ihMSCs and OEhMSC1 monolayers but not in the case of the osteogenically poor OEhMSC2 cultures (bar = 150 microns). Panel b: GW9662 induces matrix deposition by ihMSCs in a dose-dependent manner. In this case, the phase contrast is adjusted to visualize particles as dark accumulations (bar = 150 microns). Panel c: exposure of ihMSCs to GW9662 and osteogenic media causes transcriptional upregulation of various collagens found in anabolic osteoid tissue, including collagens VI and XII (means with SD, n=4). Source data are provided as a Source Data file.

## Supplementary Figure 8

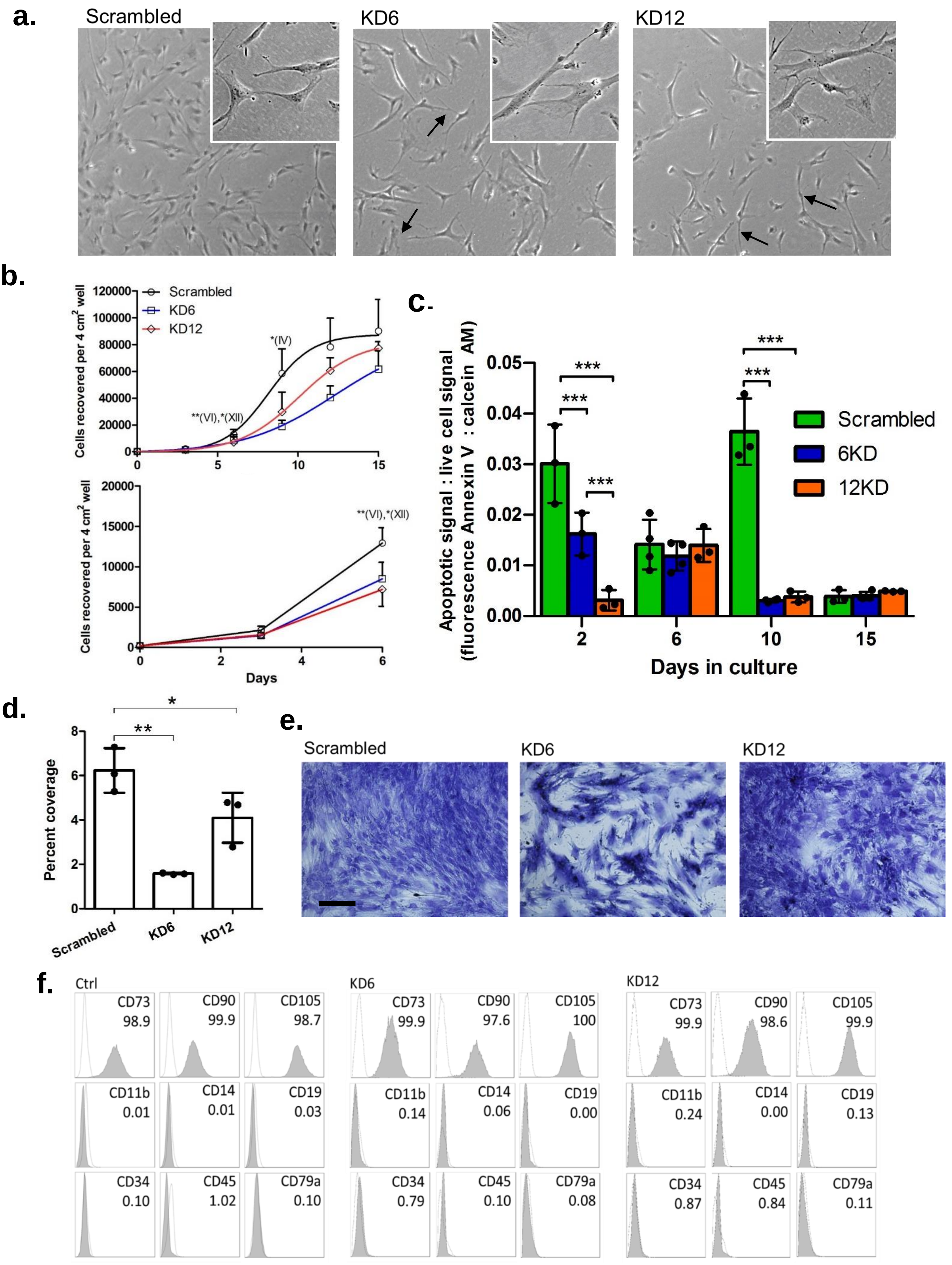


**Supplementary Figure 8: Morphology and proliferative characteristics of KD6 and KD12 hMSCs:** Panel a: phase contrast micrographs of monolayers. Low power images at 4x original magnification, bar = 200 microns. Insets at 20x original magnification, bar = 50 microns. Elongated cellular processes are arrowed. Panel b: growth curves of hMSCs over 15 days after an original seeding of 100 cells per $cm^2$. Curves for the entire duration of the experiment (left) and the first 6 days (right) are presented (n=3, presented as means with SD, analyzed by one-way ANOVA with Dunnet's post-test, p<0.05 = *, P<0.01 = **). Panel c: apoptosis levels assayed by fluorescent annexin V membrane binding levels normalized to live cell signal derived by calcein AM staining. Results are presented as the ratio of annexin V signal to calcein AM signal. Apoptosis levels are very low overall, but peak at the lag-log and log-plateau transition points in control cultures. Collagen VI or XII knockdown does not significantly increase apoptosis levels. The data are presented as means with standard deviations. The data were compared using one-way ANOVA with Tukey's post-test. *** = P<0.005, n=3. Panel d: percent coverage of colonies generated in CFU assays calculated as a percentage of the entire 154 $cm^2$ plate. Data presented and analyzed as in Panel d (* =P<0.05, *** =P<0.01, n=3). Panel e: micrographs of colonies stained with crystal violet indicating differences in morphology and cell density (bar = 100 microns). Panel f: flow cytometry indicates no significant changes in mesenchymal immunophenotype. Source data are provided as a Source Data file.

## Supplementary Figure 9

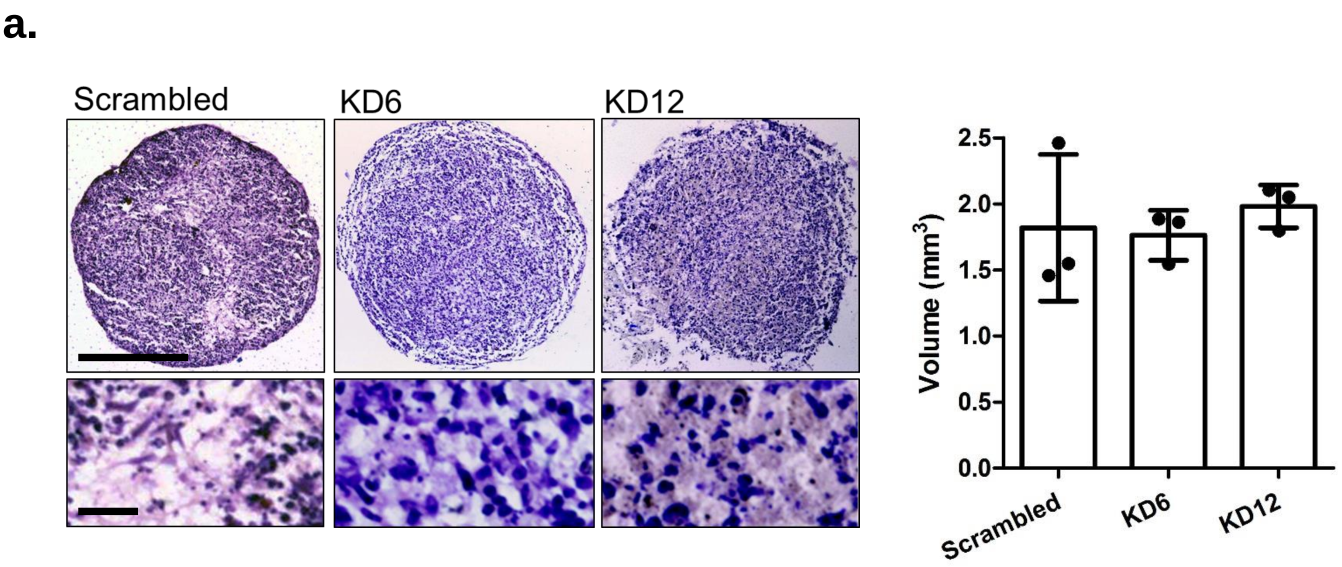


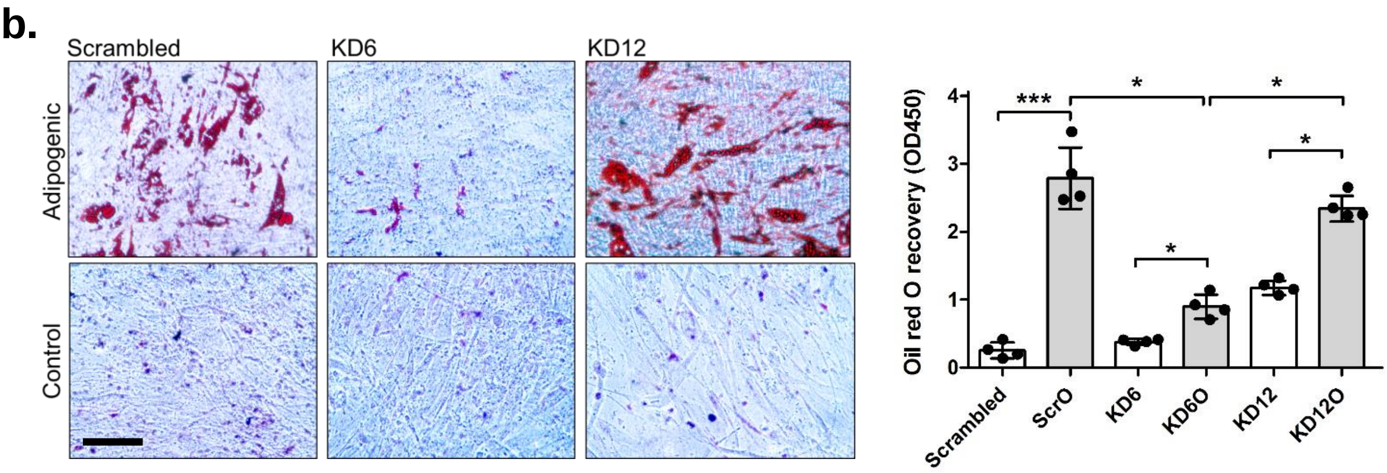


**Supplementary Figure 9: Chondrogenic and adipogenic differentiation of KD6 and KD12 hMSCs:** Panel a: chondrogenic differentiation of KD hMSCs. Micromass pellets were incubated in chondrogenic media for 21 days followed by histological sectioning and toluidine blue staining. Low power (above, bar = 1 mm) and high power (below, bar = 50 microns) micrographs are presented. Sulphated proteoglycans indicative of cartilage stains purple. Panel b: adipogenic differentiation of KD hMSCs. Micrographs of monolayers of KD hMSCs (left) incubated in the presence of adipogenic media for 21 days, then fixed and stained with oil red O to visualize fat deposits. Control cultures were incubated in CCM without adipogenic supplementation (bar = 200 microns). Quantification of staining by alcohol extraction and spectrophotometric quantification (right) for controls (open bars) and differentiated (grey bars) cultures. Statistics: the data are presented as means with standard deviations. The data were compared using one-way ANOVA with Tukey's post-test. * = $P<0.05$, *** = $P<0.005$, n=3 for panel a, n=4 for panel b. Source data are provided as a Source Data file.

## Supplementary Figure 10

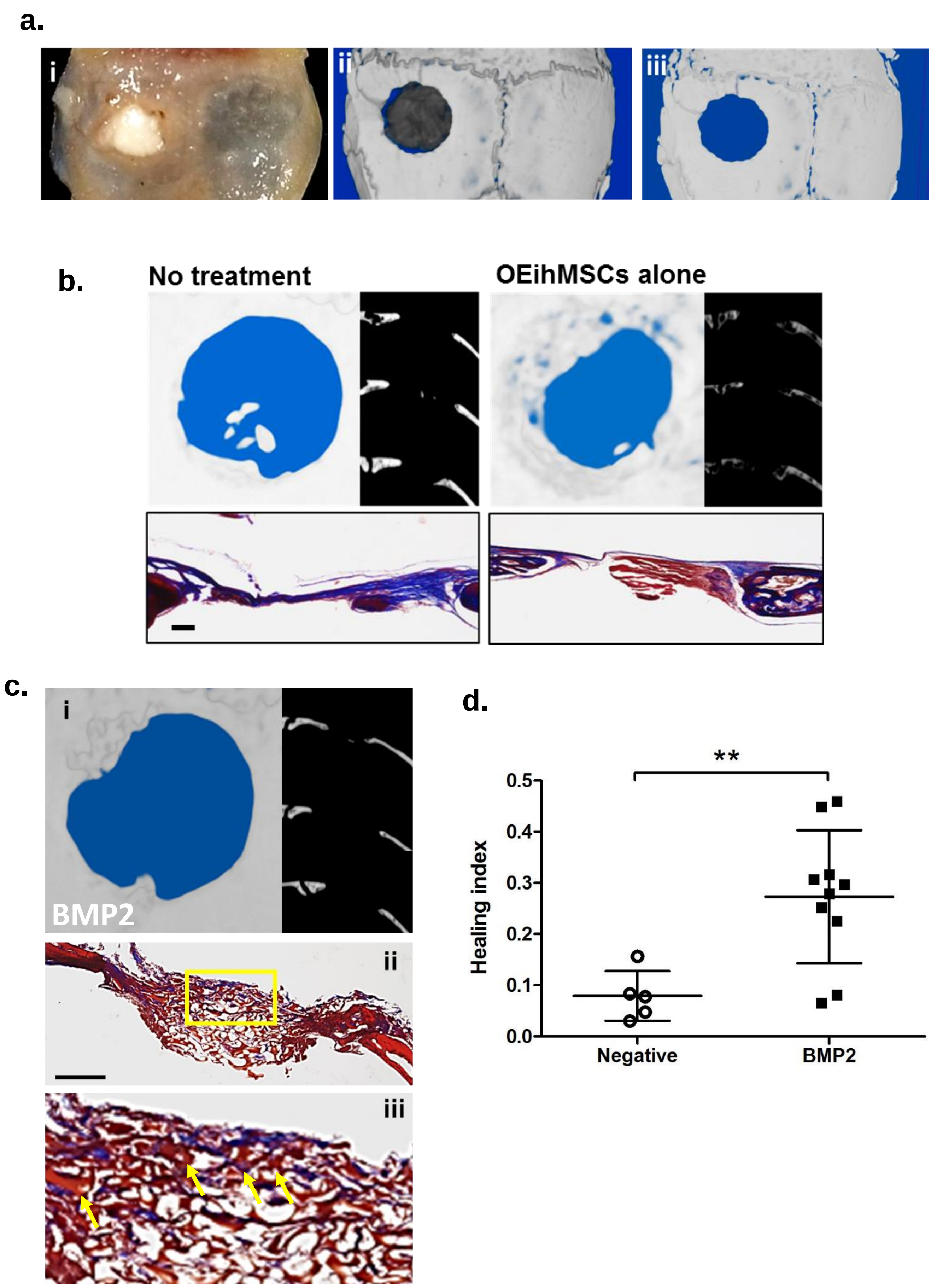


**Supplementary Figure 10: Controls and for *in vivo* experiments presented in Figure 6:** Panel a: photograph (i) of ihOCM positioned in a 4 mm murine calvarial defect with corresponding microCT rendering (ii) thresholded to detect bone only (white) and soft tissue thresholding (grey). Panel a-iii is the same as in a-ii, but only thresholded for bone detection. Under the conditions of the microCT scans, ihOCM is initially radiolucent. Panel b: microCT reconstructions (above left), axial cross-sections (above right) and Masson's trichrome stained sections of control calvarial specimens after 4 weeks of healing (bar = 250 microns). Panel c: modified scan thresholding reveals BMP2 mediated deposition of immature bone tissue. (i) microCT reconstruction (above left), axial cross-sections (above right), (ii) Masson's trichrome stained sections after 4 weeks of healing (bar = 250 microns), (iii) magnified image of region in ii (yellow box) revealing diffuse areas of bone deposition (arrowed). Panel d: healing indices calculated with modified thresholding to detect primitive osteoid demonstrate presence in some of the specimens treated with BMP2. The data (n=5 for negative, n=10 for BMP2) presented with mean (horizontal line) and SD (error bars), compared using a one-tailed Student's t-test, * = p<0.05. Source data are provided as a Source Data file.

## Supplementary Figure 11

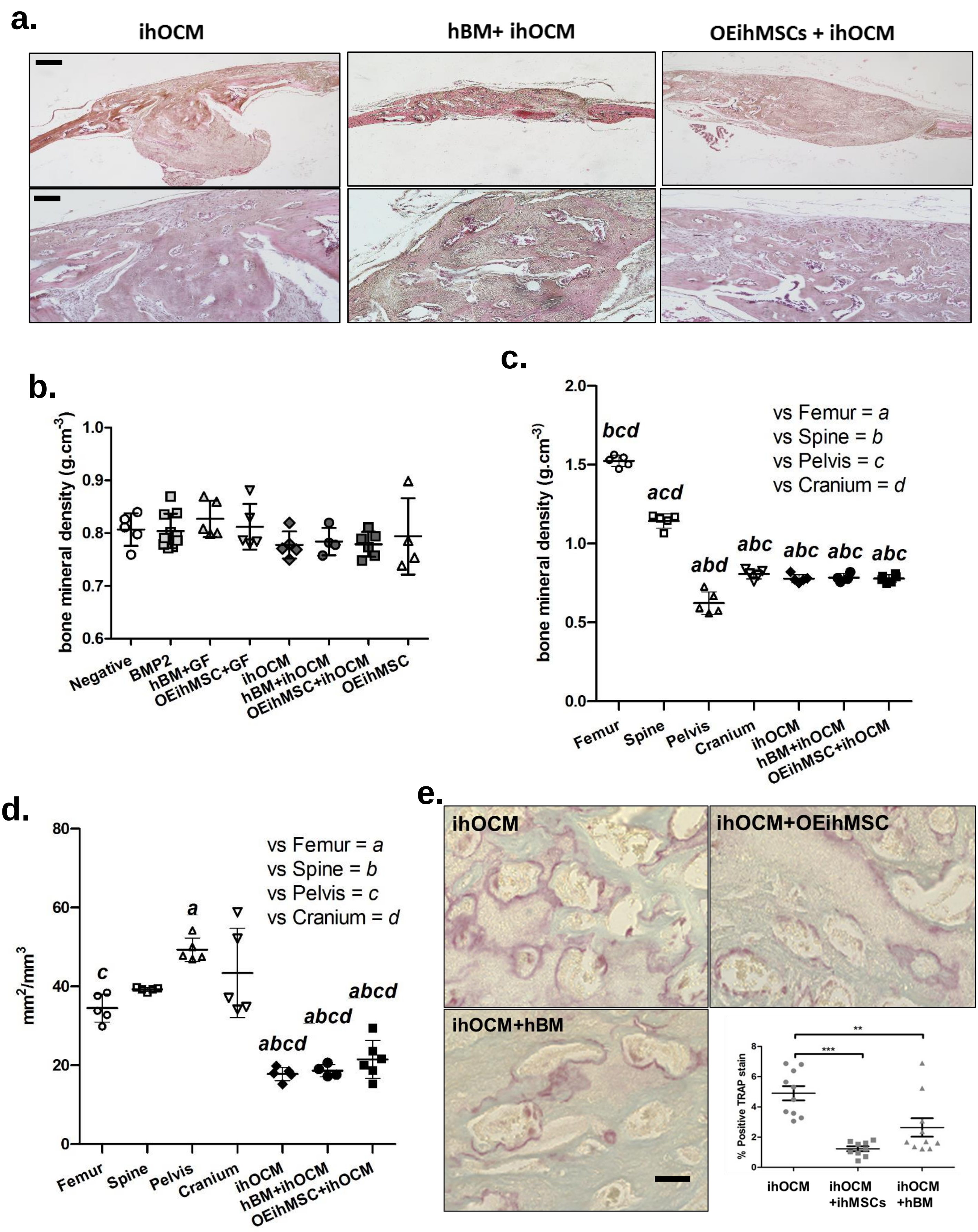


**Supplementary Figure 11: Supplemental data for in vivo experiments presented in Figure 6:** Panel a: hematoxylin and eosin stained sections at low power (above) after 4 weeks of healing (bar = 250 microns) and high power (below) of healed specimens indicating newly formed bone (bar = 75 microns). Panel b: bone mineral density at healing defect sites. Panel c: bone mineral density comparisons of the healed defects that received ihOCM and selected mature bones from various sites. Panel d: as in panel c, but for surface:volume ratio. Panel e: TRAP staining (red) for osteoclast activity at healed defect sites that contained ihOCM. Quantification of TRAP staining from randomly acquired images taken from sections at the center of each defect. Results presented as a percentage of the overall surface area measured (bar = 50 microns). Statistics: the data are presented as single measurements and means (horizontal lines) with standard deviations (vertical bars). In panel c and d, the legend refers to statistical comparisons that resulted in $p<0.005$ or $p<0.01$ (underline) with one-sided ANOVA, no differences were found between measurements in panel b. For panels b-d, all groups n=5, except ihM + hBM, n=4 and ihM + OEihMSC, n=6. For panel e, n=8 measurements between 2 specimens. Source data are provided as a Source Data file.

**Supplementary Table 1:** Primer sequences and PCR conditions used in this study. RTPrimerDB refers to the http://www.rtprimerdb.org/ database.

| Target | Sequence | Reference / notes |
|---|---|---|
| GAPDH | FOR CTCTCTGCTCCTCCTGTTCGAC<br>REV TGAGCGATGTGGCTCGGCT | 1 |
| collagen I | FOR GAACGCGTGTCATCCCTTGT<br>REV GAACGAGGTAGTCTTTCAGCAACA | RTPrimerDB ID_1089 |
| collagen III | FOR GGGAACAACTTGATGGTGCTACT<br>REV TCAGACATGAGAGTGTTTGTGCAA | RTPrimerDB ID_4463 |
| collagen V | FOR CACAACTTGCCTGATGGAATAACA<br>REV GCAGGGTACAGCTGCTTGGT | RTPrimerDB ID_1091 |
| collagen VI | FOR CCATCGTGCGCAGCC<br>REV TGCGCCGACTCGTGC | 2 |
| collagen X | FOR AATGCCTGTGTCTGCTTTTAC<br>REV ACAAGTAAAGATTCCAGTCCT | 3 |
| collagen XI | FOR GACTATCCCCTCTTCAGAACTGTTAAC<br>REV CTTCTATCAAGTGGTTTCGTGGTTT | RTPrimerDB ID_1736 |
| collagen XII | FOR CTTCCATTGAGGCAGAAGTT<br>REV AGACACAAGAGCAGCAATGA | 4 |
| collagen XV | FOR CGTGTTAGAGATGGCTGGA<br>REV GTTTGGTGGAGGCAGAAG | 5 |
| collagen XIV | FOR TCCGAGGAATGGTATAACCGG<br>REV TGGACCAGGAACACTGACAGG | 6 |
| collagen XXI | FOR GCGCAGGTCTTGCTCGGGTT<br>REV CTGGTGCTCCGGGGCAGGAT | 2 |
| runx2 | FOR GCAAGGTTCAACGATCTGAGA<br>REV TCCCCGAGGTCCATCTACTG | 7 |
| alkaline phosphatase | FOR GACCCTTGACCCCCACAAT<br>REV GCTCGTACTGCATGTCCCCT | 8 |
| osteocalcin | FOR TGAGAGCCCTCACACTCC<br>REV CGCCTGGGTCTCTTCACTAC | 9 |
| PPAR gamma | FOR CACAAGAACAGATCCAGTGGTTGCAG<br>REV AATAATAAGGTGGAGATGCAGGCTCC | RTPrimerDB ID_2420 |
| RXR alpha | REV TTCGCTAAGCTCTTGCTC<br>FOR ATAAGGAAGGTGTCAATGGG | RTPrimerDB ID_2520 |
| FABP4 | FOR TCAGTGTGAATGGGGATGTGA<br>REV TCAACGTCCCTTGGCTTATGC | RTPrimerDB ID_1965 |
| lipoprotein lipase | FOR GGAATGTATGAGAGTTGGGT<br>REV GGGCTTCTGCATACTCAAAG | 10 |
| collagen II | FOR CAACACTGCCAACG TCCAGAT<br>REV GTGGTAGGTGATGTTCT | 8 |
| COMP | FOR GACAGTGATGGCGATGGTAT<br>REV GTCATTGTCGTCGTCGTCGT | 11 |
| SOX5 | FOR GTGGCTGTTGTGAATAGTCT<br>REV CCATCATGGCATGGCTAAAT | RTPrimerDB ID_8554 |

**Supplementary Table 2:** Proteins shared in matrix derived from osteogenically enhanced MSCs generated in absence of GW9662 treatment.

| Present in all matrices. | |
|---|---|
| **ID** | **Protein name** |
| A0A087WTA8 | Collagen alpha-2(I) chain |
| P08123 | Collagen alpha-2(I) chain (Alpha-2 type I collagen) |
| P05997 | Collagen alpha-2(V) chain |
| A0A087WWY3 | Filamin-A |
| P21333 | Filamin-A (FLN-A) (Actin-binding protein 280) (ABP-280) (Alpha-filamin) (Endothelial actin-binding protein) (Filamin-1) (Non-muscle filamin) |
| P02452 | Collagen alpha-1(I) chain (Alpha-1 type I collagen) |
| P12111-4 | Collagen alpha-3(VI) chain |
| E7ENL6 | Collagen alpha-3(VI) chain |
| O43854-2 | EGF-like repeat and discoidin I-like domain-containing protein 3 (Developmentally-regulated endothelial cell locus 1 protein) (Integrin-binding protein DEL1) |
| Q96B60 | 5’-nucleotidase (5’-nucleotidase, ecto (CD73), isoform CRA_c) (NT5E protein) |
| O43854 | EGF-like repeat and discoidin I-like domain-containing protein 3 (Developmentally-regulated endothelial cell locus 1 protein) (Integrin-binding protein DEL1) |
| P12111 | Collagen alpha-3(VI) chain |
| P12111-2 | Collagen alpha-3(VI) chain |
| P21333-2 | Filamin-A (FLN-A) (Actin-binding protein 280) (ABP-280) (Alpha-filamin) (Endothelial actin-binding protein) (Filamin-1) (Non-muscle filamin) |
| P21589 | 5’-nucleotidase (5’-NT) (EC 3.1.3.5) (Ecto-5’-nucleotidase) (CD antigen CD73) |
| P21589-2 | 5’-nucleotidase (5’-NT) (EC 3.1.3.5) (Ecto-5’-nucleotidase) (CD antigen CD73) |

| Present in OEhMSC1 and OEhMSC2 | |
|---|---|
| **ID** | **Protein name** |
| P02751-9 | Fibronectin (FN) (Cold-insoluble globulin) (CIG) [Cleaved into: Anastellin; Ugl-Y1; Ugl-Y2; Ugl-Y3] |
| P02751-14 | Fibronectin (FN) (Cold-insoluble globulin) (CIG) [Cleaved into: Anastellin; Ugl-Y1; Ugl-Y2; Ugl-Y3] |
| P02751-4 | Fibronectin (FN) (Cold-insoluble globulin) (CIG) [Cleaved into: Anastellin; Ugl-Y1; Ugl-Y2; Ugl-Y3] |
| P02751-10 | Fibronectin (FN) (Cold-insoluble globulin) (CIG) [Cleaved into: Anastellin; Ugl-Y1; Ugl-Y2; Ugl-Y3] |
| P02751-13 | Fibronectin (FN) (Cold-insoluble globulin) (CIG) [Cleaved into: Anastellin; Ugl-Y1; Ugl-Y2; Ugl-Y3] |
| P02751-8 | Fibronectin (FN) (Cold-insoluble globulin) (CIG) [Cleaved into: Anastellin; Ugl-Y1; Ugl-Y2; Ugl-Y3] |
| P02751-3 | Fibronectin (FN) (Cold-insoluble globulin) (CIG) [Cleaved into: Anastellin; Ugl-Y1; Ugl-Y2; Ugl-Y3] |

| Present in OEhMSC2 and OEihMSC | |
|---|---|
| **ID** | **Protein name** |
| G5E971 | Matrix metallopeptidase 13 (Collagenase 3) |
| P45452 | Collagenase 3 (EC 3.4.24.-) (Matrix metalloproteinase-13) (MMP-13) |

| Present in OEhMSC1 and OEihMSC | |
|---|---|
| **ID** | **Protein name** |
| P05556-3 | Integrin beta-1 (Fibronectin receptor subunit beta) (Glycoprotein IIa) (GPIIA) (VLA-4 subunit beta) (CD antigen CD29) |
| P12110-2 | Collagen alpha-2(VI) chain |
| A0A087X0S5 | Collagen alpha-1(VI) chain |
| P05556-2 | Integrin beta-1 (Fibronectin receptor subunit beta) (Glycoprotein IIa) (GPIIA) (VLA-4 subunit beta) (CD antigen CD29) |
| P05556-4 | Integrin beta-1 (Fibronectin receptor subunit beta) (Glycoprotein IIa) (GPIIA) (VLA-4 subunit beta) (CD antigen CD29) |
| Q99715-2 | Collagen alpha-1(XII) chain |
| P12109 | Collagen alpha-1(VI) chain |
| P29400 | Collagen alpha-5(IV) chain |

**Supplementary Table 3:** Proteins shared in matrix derived from osteogenically enhanced MSCs generated with GW9662 treatment.

| Present in all matrices. | |
|---|---|
| **ID** | **Protein name** |
| A0A087WTA8 | Collagen alpha-2(I) chain (Alpha-2 type I collagen) |
| P08123 | Collagen alpha-2(I) chain (Alpha-2 type I collagen) |
| P02751-9 | Fibronectin (FN) (Cold-insoluble globulin) (CIG) [Cleaved into: Anastellin; Ugl-Y1; Ugl-Y2; Ugl-Y3] |
| P05997 | Collagen alpha-2(V) chain |
| P02458 | Collagen alpha-1(II) chain (Alpha-1 type II collagen) [Cleaved into: Collagen alpha-1(II) chain; Chondrocalcin] |
| P02751-14 | Fibronectin (FN) (Cold-insoluble globulin) (CIG) [Cleaved into: Anastellin; Ugl-Y1; Ugl-Y2; Ugl-Y3] |
| P02452 | Collagen alpha-1(I) chain (Alpha-1 type I collagen) |
| P12111-4 | Collagen alpha-3(VI) chain |
| E7ENL6 | Collagen alpha-3(VI) chain |
| P05556-3 | Integrin beta-1 (Fibronectin receptor subunit beta) (Glycoprotein IIa) (GPIIA) (VLA-4 subunit beta) (CD antigen CD29) |
| P02751-4 | Fibronectin (FN) (Cold-insoluble globulin) (CIG) [Cleaved into: Anastellin; Ugl-Y1; Ugl-Y2; Ugl-Y3] |
| P12110-2 | Collagen alpha-2(VI) chain |
| A0A087X0S5 | Collagen alpha-1(VI) chain |
| P12111 | Collagen alpha-3(VI) chain |
| P12111-2 | Collagen alpha-3(VI) chain |
| P05556-2 | Integrin beta-1 (Fibronectin receptor subunit beta) (Glycoprotein IIa) (GPIIA) (VLA-4 subunit beta) (CD antigen CD29) |
| P05556-4 | Integrin beta-1 (Fibronectin receptor subunit beta) (Glycoprotein IIa) (GPIIA) (VLA-4 subunit beta) (CD antigen CD29) |
| P12109 | Collagen alpha-1(VI) chain |
| P02751-10 | Fibronectin (FN) (Cold-insoluble globulin) (CIG) [Cleaved into: Anastellin; Ugl-Y1; Ugl-Y2; Ugl-Y3] |
| P02751-13 | Fibronectin (FN) (Cold-insoluble globulin) (CIG) [Cleaved into: Anastellin; Ugl-Y1; Ugl-Y2; Ugl-Y3] |
| P02751-8 | Fibronectin (FN) (Cold-insoluble globulin) (CIG) [Cleaved into: Anastellin; Ugl-Y1; Ugl-Y2; Ugl-Y3] |
| P02751-3 | Fibronectin (FN) (Cold-insoluble globulin) (CIG) [Cleaved into: Anastellin; Ugl-Y1; Ugl-Y2; Ugl-Y3] |
| P21589 | 5’-nucleotidase (5’-NT) (EC 3.1.3.5) (Ecto-5’-nucleotidase) (CD antigen CD73) |
| P21589-2 | 5’-nucleotidase (5’-NT) (EC 3.1.3.5) (Ecto-5’-nucleotidase) (CD antigen CD73) |
| P35555 | Fibrillin-1 [Cleaved into: Asprosin] |

| Present in OEhMSC1 and OEhMSC2 | |
|---|---|
| **ID** | **Protein name** |
| O43854-2 | EGF-like repeat and discoidin I-like domain-containing protein 3 (Developmentally-regulated endothelial cell locus 1 protein) (Integrin-binding protein DEL1) |
| Q96B60 | 5’-nucleotidase (5’-nucleotidase, ecto (CD73), isoform CRA_c) (NT5E protein) |
| O43854 | EGF-like repeat and discoidin I-like domain-containing protein 3 (Developmentally-regulated endothelial cell locus 1 protein) (Integrin-binding protein DEL1) |

**Supplementary Table 4:** P-values and confidence intervals for calvarial defect HI measurements One way ANOVA. *P<0.05, ** P<0.01, ***P<0.005, *ns* not significant (P>0.05).

| Tukey's Multiple Comparison Test | P < 0.05? | Summary | 95% CI of diff |
|---|---|---|---|
| Negative vs BMP2 | Yes | * | -0.84 to 0.35 |
| Negative vs hBM+GF | No | ns | -0.92 to 0.39 |
| Negative vs OEihMSC+GF | No | ns | -1.02 to 0.29 |
| Negative vs ihOCM | Yes | *** | -3.21 to -1.89 |
| Negative vs hBM+ihOCM | Yes | *** | -2.49 to -1.10 |
| Negative vs OEihMSC+ihOCM | Yes | *** | -1.83 to -0.575 |
| Negative vs OEihMSC | No | ns | -1.01 to 0.3873 |
| BMP2 vs hBM+GF | No | ns | -0.6120 to 0.57 |
| BMP2 vs OEihMSC+GF | No | ns | -0.71 to 0.47 |
| BMP2 vs ihOCM | Yes | *** | -2.89 to -1.71 |
| BMP2 vs hBM+ihOCM | Yes | *** | -2.19 to -0.92 |
| BMP2 vs OEihMSC+ihOCM | Yes | *** | -1.52 to -0.39 |
| BMP2 vs OEihMSC | No | ns | -0.70 to 0.57 |
| hBM+GF vs OEihMSC+GF | No | ns | -0.75 to 0.56 |
| hBM+GF vs ihOCM | Yes | *** | -2.94 to -1.63 |
| hBM+GF vs hBM+ihOCM | Yes | *** | -2.23 to -0.84 |
| hBM+GF vs OEihMSC+ihOCM | Yes | *** | -1.57 to -0.31 |
| hBM+GF vs OEihMSC | No | ns | -0.74 to 0.65 |
| OEihMSC+GF vs ihOCM | Yes | *** | -2.84 to -1.53 |
| OEihMSC+GF vs hBM+ihOCM | Yes | *** | -2.13 to -0.74 |
| OEihMSC+GF vs OEihMSC+ihOCM | Yes | ** | -1.47 to -0.21 |
| OEihMSC+GF vs OEihMSC | No | ns | -0.64 to 0.75 |
| ihOCM vs hBM+ihOCM | Yes | * | 0.05 to 1.44 |
| ihOCM vs OEihMSC+ihOCM | Yes | *** | 0.71 to 1.97 |
| ihOCM vs OEihMSC | Yes | *** | 1.54 to 2.93 |
| hBM+ihOCM vs OEihMSC+ihOCM | No | ns | -0.07 to 1.27 |
| hBM+ihOCM vs OEihMSC | Yes | *** | 0.76 to 2.22 |
| OEihMSC+ihOCM vs OEihMSC | Yes | ** | 0.224to 1.56 |

**Supplementary Table 5:** P-values and confidence intervals for calvarial defect surface to volume ratios. One way ANOVA. *P<0.05, ** P<0.01, ***P<0.005, *ns* not significant (P>0.05).

| Tukey's Multiple Comparison Test | P < 0.05? | Summary | 95% CI of diff |
|---|---|---|---|
| Negative vs BMP2 | Yes | * | 1.78 to 23.05 |
| Negative vs hBM+GF | Yes | ** | 3.84 to 27.96 |
| Negative vs OEihMSC+GF | Yes | ** | 4.91 to 29.02 |
| Negative vs ihOCM | Yes | *** | 13.53 to 37.64 |
| Negative vs hBM+ihOCM | Yes | *** | 11.97 to 37.54 |
| Negative vs OEihMSC+ihOCM | Yes | *** | 10.37 to 33.46 |
| Negative vs OEihMSC | Yes | * | 1.35 to 26.93 |
| BMP2 vs hBM+GF | No | ns | -7.15 to 14.11 |
| BMP2 vs OEihMSC+GF | No | ns | -6.08 to 15.18 |
| BMP2 vs ihOCM | Yes | ** | 2.53 to 23.80 |
| BMP2 vs hBM+ihOCM | Yes | * | 0.88 to 23.79 |
| BMP2 vs OEihMSC+ihOCM | No | ns | -0.55 to 19.54 |
| BMP2 vs OEihMSC | No | ns | -9.73 to 13.18 |
| hBM+GF vs OEihMSC+GF | No | ns | -10.99 to 13.12 |
| hBM+GF vs ihOCM | No | ns | -2.37 to 21.74 |
| hBM+GF vs hBM+ihOCM | No | ns | -3.93 to 21.64 |
| hBM+GF vs OEihMSC+ihOCM | No | ns | -5.53 to 17.56 |
| hBM+GF vs OEihMSC | No | ns | -14.55 to 11.03 |
| OEihMSC+GF vs ihOCM | No | ns | -3.44 to 20.67 |
| OEihMSC+GF vs hBM+ihOCM | No | ns | -4.99 to 20.58 |
| OEihMSC+GF vs OEihMSC+ihOCM | No | ns | -6.59 to 16.49 |
| OEihMSC+GF vs OEihMSC | No | ns | -15.61 to 9.96 |
| ihOCM vs hBM+ihOCM | No | ns | -13.62 to 11.96 |
| ihOCM vs OEihMSC+ihOCM | No | ns | -15.21 to 7.87 |
| ihOCM vs OEihMSC | No | ns | -24.23 to 1.34 |
| hBM+ihOCM vs OEihMSC+ihOCM | No | ns | -15.14 to 9.46 |
| hBM+ihOCM vs OEihMSC | No | ns | -24.09 to 2.86 |
| OEihMSC+ihOCM vs OEihMSC | No | ns | -20.08 to 4.53 |

## References.


1 Carraro, G., Albertin, G., Forneris, M. & Nussdorfer, G. G. Similar sequence-free amplification of human glyceraldehyde-3-phosphate dehydrogenase for real time RT-PCR applications. *Mol Cell Probes* **19**, 181-186, doi:S0890-8508(04)00106-9 [pii]10.1016/j.mcp.2004.11.004 (2005).

2 Zeitouni, S. *et al.* Human mesenchymal stem cell-derived matrices for enhanced osteoregeneration. *Sci Transl Med* **4**, 132ra155, doi:4/132/132ra55 [pii]10.1126/scitranslmed.3003396 (2012).

3 Alaseem, A. M. *et al.* Naproxen induces type X collagen expression in human bone-marrow-derived mesenchymal stem cells through the upregulation of 5-lipoxygenase. *Tissue Eng Part A* **21**, 234-245, doi:10.1089/ten.TEA.2014.0148 (2015).

4 Nemoto, T., Kajiya, H., Tsuzuki, T., Takahashi, Y. & Okabe, K. Differential induction of collagens by mechanical stress in human periodontal ligament cells. *Arch Oral Biol* **55**, 981-987, doi:S0003-9969(10)00232-3 [pii]10.1016/j.archoralbio.2010.08.004 (2010).

5 Lisignoli, G. *et al.* Gene array profile identifies collagen type XV as a novel human osteoblast-secreted matrix protein. *J Cell Physiol* **220**, 401-409, doi:10.1002/jcp.21779 (2009).

6 Schmidt, A., Lorkowski, S., Seidler, D., Breithardt, G. & Buddecke, E. TGF-beta1 generates a specific multicomponent extracellular matrix in human coronary SMC. *Eur J Clin Invest* **36**, 473-482, doi:ECI1658 [pii]10.1111/j.1365-2362.2006.01658.x (2006).

7 Schaap-Oziemlak, A. M. *et al.* MicroRNA hsa-miR-135b regulates mineralization in osteogenic differentiation of human unrestricted somatic stem cells. *Stem Cells Dev* **19**, 877-885, doi:10.1089/scd.2009.0112 (2010).

8 Zhao, Q. *et al.* MSCs derived from iPSCs with a modified protocol are tumor-tropic but have much less potential to promote tumors than bone marrow MSCs. *Proc Natl Acad Sci U S A* **112**, 530-535, doi:10.1073/pnas.1423008112 (2015).

9 Salem, O. *et al.* Naproxen affects osteogenesis of human mesenchymal stem cells via regulation of Indian hedgehog signaling molecules. *Arthritis Res Ther* **16**, R152, doi:10.1186/ar4614 (2014).

10 Gafni, Y. *et al.* Gene therapy platform for bone regeneration using an exogenously regulated, AAV-2-based gene expression system. *Mol Ther* **9**, 587-595, doi:10.1016/j.ymthe.2003.12.009S1525001603004131 [pii] (2004).

11 Chen, X. *et al.* Chondrogenic differentiation of umbilical cord-derived mesenchymal stem cells in type I collagen-hydrogel for cartilage engineering. *Injury* **44**, 540-549, doi:10.1016/j.injury.2012.09.024 (2013).
