# Supplementary material for: Characterization of a pluripotent stem cell-derived matrix with powerful osteoregenerative capabilities
Source: Nat Commun. 2020 Jun 15;11:3025. doi: 10.1038/s41467-020-16646-2 (PMC7295745; doi:10.1038/s41467-020-16646-2)
Supplement: Supplementary file 3 — Reporting Summary [file 41467_2020_16646_MOESM3_ESM.pdf]

nature research

Corresponding author(s): Gregory, Carl A.

Last updated by author(s): May 6, 2020

Reporting Summary

Nature Research wishes to improve the reproducibility of the work that we publish. This form provides structure for consistency and transparency in reporting. For further information on Nature Research policies, see Authors & Referees and the Editorial Policy Checklist .

Statistics

For all statistical analyses, confirm that the following items are present in the figure legend, table legend, main text, or Methods section.

n/a	Confirmed	
☐	☒	The exact sample size (*n*) for each experimental group/condition, given as a discrete number and unit of measurement
☐	☒	A statement on whether measurements were taken from distinct samples or whether the same sample was measured repeatedly
☐	☒	The statistical test(s) used AND whether they are one- or two-sided<br>*Only common tests should be described solely by name; describe more complex techniques in the Methods section.*
☒	☐	A description of all covariates tested
☒	☐	A description of any assumptions or corrections, such as tests of normality and adjustment for multiple comparisons
☐	☒	A full description of the statistical parameters including central tendency (e.g. means) or other basic estimates (e.g. regression coefficient) AND variation (e.g. standard deviation) or associated estimates of uncertainty (e.g. confidence intervals)
☐	☒	For null hypothesis testing, the test statistic (e.g. *F*, *t*, *r*) with confidence intervals, effect sizes, degrees of freedom and *P* value noted<br>*Give P values as exact values whenever suitable.*
☒	☐	For Bayesian analysis, information on the choice of priors and Markov chain Monte Carlo settings
☒	☐	For hierarchical and complex designs, identification of the appropriate level for tests and full reporting of outcomes
☒	☐	Estimates of effect sizes (e.g. Cohen's *d*, Pearson's *r*), indicating how they were calculated

Our web collection on statistics for biologists contains articles on many of the points above.

Software and code

Policy information about availability of computer code

Data collection	CXP (Becton Coulter) for flow cytometry acquisition.
Data analysis	CTAn, NRecon, CTvol (Bruker) for processing of micro computed tomography. Rstudio (v1.1.435), ggplot (3.0.0), dplyr (0.7.6) and reshape (1.4.3) for processing PCR data. Proteome Discoverer 2 software (Thermo) and Rstudio (v1.1.435) for proteomic data.

For manuscripts utilizing custom algorithms or software that are central to the research but not yet described in published literature, software must be made available to editors/reviewers. We strongly encourage code deposition in a community repository (e.g. GitHub). See the Nature Research guidelines for submitting code & software for further information.

Data

Policy information about availability of data

All manuscripts must include a data availability statement. This statement should provide the following information, where applicable:

- Accession codes, unique identifiers, or web links for publicly available datasets
- A list of figures that have associated raw data
- A description of any restrictions on data availability

Data availability statement: Proteomic data sets accessible via the the PRIDE Database (http://www.ebi.ac.uk/pride) accession number: PXD016017. Complete western blot bitmaps and raw numerical data are available in a raw datafile in accordance with journal policies.

Field-specific reporting

Please select the one below that is the best fit for your research. If you are not sure, read the appropriate sections before making your selection.

Life sciences study design

All studies must disclose on these points even when the disclosure is negative.

Sample size	Sample sizes determined by post hoc power analysis where values are quantitative. Where values are qualitative, specimens were processed in triplicate based on previous studies.
Data exclusions	A Grubbs outlier analysis was performed in each case. No outliers were detected.
Replication	All in vitro experiments were carried out on at least two separate occasions, and key experiments were repeated by collaborators at alternate sites. Replicates yielded the same outcome in each case. Animal studies were performed as thoroughly as possible with appropriate group size calculations, and redundant controls in accordance with IACUC requirements. Key in vivo experiments (such as efficacy of ihOCM, collagen 6 and 12 KD experiments) were were repeated independently to confirm robustness of major findings, but comprehensive repetition was limited by IACUC policies.
Randomization	Mice were randomized into test groups upon delivery. Complex design of studies did not permit prior randomization, but data were interpreted blindly by third party collaborators/authors.
Blinding	Analyses of calvarial defects were performed using coded data-sets. In vitro experiments were not randomized due to the complexity of the study design, but third party validation of our interpretations was performed by collaborating authors without the initial identification of experimental groups.

Reporting for specific materials, systems and methods

We require information from authors about some types of materials, experimental systems and methods used in many studies. Here, indicate whether each material, system or method listed is relevant to your study. If you are not sure if a list item applies to your research, read the appropriate section before selecting a response.

Materials & experimental systems

n/a	Involved in the study	
☐	☒	Antibodies
☐	☒	Eukaryotic cell lines
☒	☐	Palaeontology
☐	☒	Animals and other organisms
☒	☐	Human research participants
☒	☐	Clinical data

Methods

n/a	Involved in the study	
☒	☐	ChIP-seq
☐	☒	Flow cytometry
☒	☐	MRI-based neuroimaging

Antibodies

Antibodies used	Fluorophore-tagged antibodies (Becton Dickinson, Franklin Lakes, NJ or Beckman Coulter) CD11b (clone BEAR1, #IMO53OU), CD14 (RMO52, #IMO65OU), CD19 (J3-119,#IM1285U), CD34 (581, #IM1871U), CD45 (J.33, #IM0782U), CD73 (AD2, #B68176), CD79a (HM47, #IM2221U), CD90 (Thy-1/310, #IM1839U), CD105 (TEA3, #B76299), and HLA-DP, DQ, DR (9-49, #18142), and appropriate isotype controls (FITC: B76299, 6603864, 6603853, 6603855, B36627. PE: A09141, IM0670U).<br>Cellular fractionation blots were probed using mouse anti-human GAPDH (clone 6C5, Chemicon International, Temecula, CA. #MAB374), mouse anti-human beta catenin (clone 5H10, Chemicon. #MAB2081), mouse anti-human GSK3beta clone 3D10, Abcam, Cambridge, UK. #ab93926), mouse anti-human PPARgamma (clone 1E6A1, Thermo Fisher. #MA5-15417). Secondary antibody was goat anti-mouse IgG conjugate (Thermo Fisher, #G21040).<br>Immunoblotting for collagen VI and XII was performed on whole cell lysates using rabbit-anti human type VI collagen (NBP159126, Novus Biologicals, Littleton, CO), rabbit-anti human type XII collagen (NBP1-88062, Novus), goat anti-rabbit IgG-peroxidase conjugate (#sc2004, Santa Cruz, Dallas, TX).
Validation	FLOW CYTOMETRY: All antibodies were validated by quality control statements on websites and on batch analysis statements. Validation data (and clinical regulatory status - all are clinically validated for diagnostic purposes) for all 17 flow cytometry viewed on the Beckman website (https://www.beckman.com/reagents/coulter-flow-cytometry/antibodies-and-kits/single-color-antibodies).<br>WESTERN BLOTTING: All antibodies have been validated for application by the company. For GAPDH: https://www.emdmillipore.com/US/en/product/Anti-Glyceraldehyde-3-Phosphate-Dehydrogenase-Antibody-clone-6C5,MM_NF-MAB374, for BETA-CATENIN: https://www.emdmillipore.com/US/en/product/Anti-Catenin-Antibody-clone-5H10,MM_NF-MAB2081), for GSK3BETA: https://www.abcam.com/gsk3-beta-antibody-3d10-ab93926.html, for PPARGAMMA: https://www.thermofisher.com/antibody/product/PPAR-gamma-Antibody-clone-3A4A9-1E6A1-Monoclonal/MA5-15417, for GOAT anti-MOUSE, https://www.thermofisher.com/antibody/secondary/query/goat%20anti%20mouse%20secondary/filter/species/Mouse/conjugates/HRP, for COLLAGEN VI, https://www.novusbio.com/search?keywords=NBP159126, for COLLAGEN XII, https://www.novusbio.com/products/collagen-xii-alpha1-antibody_nbp1-88062, for GOAT ANTI_RABBIT, https://www.scbt.com/p/goat-anti-rabbit-igg-hrp)

All of the antibodies have been used by our group in various capacities and on various types of specimens for over 4 years.

Eukaryotic cell lines

Policy information about cell lines

Cell line source(s)	Two human bone marrow derived mesenchymal stem cell preparations and iPSC-derived MSCs originally described by Zhao et al 2014 (PMID:25548183). The bone marrow derived mesenchymal stem cells were acquired form the NIH funded mesenchymal stem cell core at Texas A&M Institute for Regenerative Medicine, validated by immunophenotype, morphology and differentiation capacity as defined by the International Society for Cell Therapy (ISCT). The iPSC-derived MSCs were generated by Fei Liu's group at the Institute using the CY2 cell line originally acquired from Cellular Dynamics International. RAW264.7 were acquired from the American Type Culture Collection.
Authentication	Human bone marrow-derived mesenchymal stem cells (hMSCs) were acquired from the Texas A&M Health Science Center Institute for Regenerative Medicine MSC distribution facility in accordance with institutionally approved protocols. The cells are pre-validated in accordance with the Dominici criteria and several additional assays (see https://medicine.tamhsc.edu/centers/irm/index.html). The iPS-derived MSCs were originally acquired from Cellular Dynamics International and MSCs derived form them were validated in Zhao et al (referenced in manuscript), in the manuscript itself, and in several studies performed by the IRM MSC core. Cells were also validated using species specific primers for known biomarkers.
Mycoplasma contamination	Tested negative
Commonly misidentified lines (See ICLAC register)	No commonly misidentified cell lines were used in the study.

Animals and other organisms

Policy information about studies involving animals; ARRIVE guidelines recommended for reporting animal research

Laboratory animals	Female immune deficient (nu/nu) nude mice at 2 months of age acquired from Jackson laboratories. Animals were housed in accordance with NIH and OLAW guidelines on IACUC approved protocols at 21-25 degC, 50 - 70% relative humidity and 12h-12h light cycle.
Wild animals	No wild animals were used in the study.
Field-collected samples	No field collected animals were used in the study.
Ethics oversight	All protocols involving animals were approved by the Texas A&M Institutional Animal Care and Use Committee (IACUC).

Note that full information on the approval of the study protocol must also be provided in the manuscript.

Flow Cytometry

Plots

Confirm that:

- [x] The axis labels state the marker and fluorochrome used (e.g. CD4-FITC).
- [x] The axis scales are clearly visible. Include numbers along axes only for bottom left plot of group (a 'group' is an analysis of identical markers).
- [x] All plots are contour plots with outliers or pseudocolor plots.
- [x] A numerical value for number of cells or percentage (with statistics) is provided.

Methodology

Sample preparation	MSCs were recovered with trypsin/EDTA and re-suspended in phosphate buffered saline (PBS, Life technologies) supplemented with 2% (v/v) FBS (Atlanta Biologicals) with fluorophore-tagged antibodies (Becton Dickinson, Franklin Lakes, NJ or Beckman Coulter) for 30 minutes on ice. Antibodies against CD11b (clone BEAR1, #IMO53OU), CD14 (RMO52, #IMO65OU), CD19 (J3-119,#IM1285U), CD34 (581, #IM1871U), CD45 (J.33, #IM0782U), CD73 (AD2, #B68176), CD79a (HM47, #IM2221U), CD90 (Thy-1/310, #IM1839U), CD105 (TEA3, #B76299), and HLA-DP, DQ, DR (9-49, #18142), and appropriate isotype controls (FITC: B76299, 6603864, 6603853, 6603855, B36627. PE: A09141, IM0670U) were used. The cells were analyzed using a Cytomics FC500 flow cytometer (Beckman Coulter) and data were processed using the manufacturer's software (CXP).
Instrument	Cytomics FC500 flow cytometer (Beckman Coulter)
Software	Data were processed using the manufacturer's software (CXP).
Cell population abundance	At least 100,000 events were recorded.

Gating strategy

Strategy defined in S2b and S2c. Readings compared to negative controls, and positive measurements at least one log above control readings. See Fig 1d and e.

☒ Tick this box to confirm that a figure exemplifying the gating strategy is provided in the Supplementary Information.
